# Supplementary material for: Host Immunosuppression Induced by Steinernema feltiae, an Entomopathogenic Nematode, through Inhibition of Eicosanoid Biosynthesis
Source: Insects. 2019 Dec 31;11(1):33. doi: 10.3390/insects11010033 (PMC7023448; doi:10.3390/insects11010033)
Supplement: Supplementary file 1 [file insects-11-00033-s001.pdf]

insects

Article

Host Immunosuppression Induced by *Steinernema feltiae*, an Entomopathogenic Nematode, through Inhibition of Eicosanoid Biosynthesis

Miltan Chandra Roy [1], Dongwoon Lee [2], and Yonggyun Kim [1,*]

[1] Department of Plant Medicals, Andong National University, Andong 36729, Korea; miltan.roy@yahoo.com
[2] School of Environmental Ecology and Tourism, Kyungpook National University, Sangju 37224, Korea; whitegrub@knu.ac.kr
* Correspondence: hosanna@anu.ac.kr; Tel.: +82-54-820-5638

Received: 10 December 2019; Accepted: 28 December 2019; Published: 31 December 2019

Supplementary Material

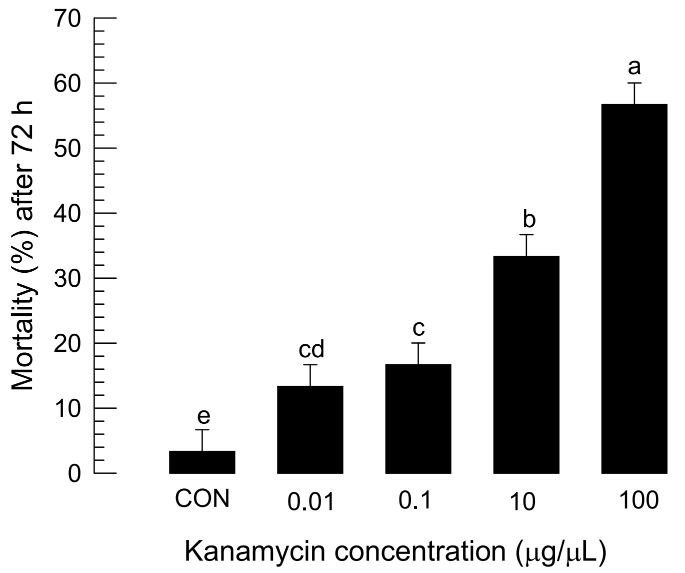

Figure S1. Screening to find the optimum concentration of kanamycin against fourth instar larva of *P. xylostella* by mortality assay. Kanamycin at indicated concentration was injected into the hemocoel. Each concentration was replicated three times. For each replicate, 10 larvae of target insects were used. Different letters above standard error bars, indicate significant differences among means at Type I error = 0.05 (LSD test).

© 2019 by the authors. Licensee MDPI, Basel, Switzerland. This article is an open access article distributed under the terms and conditions of the Creative Commons Attribution (CC BY) license (http://creativecommons.org/licenses/by/4.0/).
